# Impaired synaptic incorporation of AMPA receptors in a mouse model of fragile X syndrome

**DOI:** 10.3389/fnmol.2023.1258615

**Published:** 2023-11-09

**Authors:** Magdalena Chojnacka, Anna Beroun, Marta Magnowska, Aleksandra Stawikowska, Dominik Cysewski, Jacek Milek, Magdalena Dziembowska, Bozena Kuzniewska

**Affiliations:** ^1^Laboratory of Molecular Basis of Synaptic Plasticity, Centre of New Technologies, University of Warsaw, Warsaw, Poland; ^2^Laboratory of Neuronal Plasticity, Nencki Institute of Experimental Biology of Polish Academy of Sciences, Warsaw, Poland; ^3^Clinical Research Centre, Medical University of Bialystok, Bialystok, Poland

**Keywords:** FXS, *Fmr1* KO, GluA2, AMPA receptors, synaptic plasticity, brain

## Abstract

Fragile X syndrome (FXS) is the most common monogenetic cause of inherited intellectual disability and autism in humans. One of the well-characterized molecular phenotypes of *Fmr1* KO mice, a model of FXS, is increased translation of synaptic proteins. Although this upregulation stabilizes in adulthood, abnormalities during the critical period of plasticity have long-term effects on circuit formation and synaptic properties. Using high-resolution quantitative proteomics of synaptoneurosomes isolated from the adult, developed brains of *Fmr1* KO mice, we show a differential abundance of proteins regulating the postsynaptic receptor activity of glutamatergic synapses. We investigated the AMPA receptor composition and shuttling in adult *Fmr*1 KO and WT mice using a variety of complementary experimental strategies such as surface protein crosslinking, immunostaining of surface receptors, and electrophysiology. We discovered that the activity-dependent synaptic delivery of AMPARs is impaired in adult *Fmr1* KO mice. Furthermore, we show that *Fmr1* KO synaptic AMPARs contain more GluA2 subunits that can be interpreted as a switch in the synaptic AMPAR subtype toward an increased number of Ca^2+−^impermeable receptors in adult *Fmr1* KO synapses.

## Introduction

Fragile X syndrome (FXS) is the most common form of inherited intellectual disability and the most common single-gene cause of autism (Hagerman et al., 2005). FXS results from trinucleotide CGG-repeat expansion in the *FMR1* gene, which leads to gene methylation, silencing, and loss of Fragile X messenger ribonucleoprotein 1 (FMRP) (Yu et al., 1991). FMRP is an RNA-binding protein that regulates the translation of many synaptic proteins (Bassell and Warren, 2008; Darnell and Klann, 2013). Several studies focused on characterizing mRNAs that are associated with FMRP, and more than 1,000 FMRP-target mRNAs have been identified (Brown et al., 2001; Darnell et al., 2011).

The most commonly used animal model of FXS is *Fmr1* KO mice, which recapitulate many physiological and behavioral features of human disease (Consorthium et al., 1994). Since the establishment of the model, numerous studies have been aimed at identifying pathological mechanisms and potential therapeutic interventions in FXS. There is comprehensive literature on *Fmr1* KO mice; however, the obtained results are often inconsistent or even contradictory (reviewed in Kat et al., 2022). Many studies of *Fmr1* KO mice have been performed on young mice, identifying phenotypes during early postnatal development. However, it appears that many of the observed differences between *Fmr1* KO and WT mice are developmentally transient (reviewed in Razak et al., 2020). Moreover, the main conclusion of the research so far is that the loss of FMRP results in brain region and cell-type-specific effects (Larson et al., 2005; Sawicka et al., 2019; Donnard et al., 2022). FXS is a neurodevelopmental disorder, and the FMRP level is developmentally regulated (Gholizadeh et al., 2015). In the mouse brain, FMRP expression peaks in the first 2 postnatal weeks and is reduced in adulthood. Therefore, it is not surprising that the absence of FMRP during the critical period of plasticity has complex and long-term effects on circuit formation, synaptogenesis, and synaptic properties.

One of the well-characterized molecular phenotypes in the *Fmr1* KO mouse brain is increased protein synthesis (Greenough et al., 2001; Osterweil et al., 2010). However, although hundreds of proteins are upregulated in young *Fmr1* KO mice, this upregulation is largely attenuated in adulthood (Tang et al., 2015). The clinical phenotype of FXS includes hyperactivity and sensory integration defects, i.e., hypersensitivity to sensory stimuli (Liu et al., 2021), indicating network hyperexcitability. Indeed, studies in *Fmr1* KO mice show hyperexcitability or an imbalance between excitation and inhibition (E/I) in neuronal networks (Contractor et al., 2015; Nelson and Valakh, 2015; Antoine et al., 2019). The synaptic E/I ratio is fine-tuned during brain development as neuronal circuits mature structurally and functionally (Chen et al., 2022). Moreover, the daily oscillations of the E/I ratio were shown in the visual cortex (Bridi et al., 2020). Recently, alterations in both neural firing rates and correlations were observed in Fmr1 KO mice across development (O'Donnell et al., 2017). Recently, there has been growing evidence that the loss of FMRP leads to different ion channel dysfunctions that may underlie the abovementioned symptoms associated with FXS (reviewed in Deng and Klyachko, 2021). Several studies have reported that the loss of FMRP can lead to abnormalities in the translational control of different subunits of ion channels (e.g., K^+^, AMPA, NMDA, and GABA) as well as in their activity or surface expression, but the results seem to be inconsistent or even contradictory and may be due to studied brain structures. AMPA receptors (AMPARs) are glutamate-gated ion channels, which are the major mediators of fast excitatory transmission in the brain. Functional AMPARs on the cell surface are homo- or hetero-tetramers, assembled from combinations of four subunits: GluA1, GluA2, GluA3, and GluA4 (Shepherd and Huganir, 2007). AMPARs are highly dynamic, shuttling between the synaptic membrane and the inside of the synapse, undergoing exocytosis, lateral diffusion to the postsynaptic density (PSD), and endocytosis (Malinow and Malenka, 2002; Diering and Huganir, 2018). Changes in the number, composition, and biophysical properties of AMPARs in the postsynaptic membrane are the main mechanisms controlling synaptic strength during various forms of synaptic plasticity (reviewed: Malinow and Malenka, 2002; Diering and Huganir, 2018). The different subunit compositions of AMPARs determine their biophysical properties and cellular trafficking (Shepherd and Huganir, 2007). In general, AMPARs are incorporated into the PSD during LTP, with the first ones recruited being GluA1 homomers. In contrast, during LTD surface, AMPARs are depleted, initially by endocytosis of GluA2-containing receptors (review Diering and Huganir, 2018). Overall, the synaptic accumulation or removal of AMPARs is a complex, tightly regulated process that affects synaptic strength and relies on subunit-specific protein interactions. Importantly, the composition of AMPAR subunits determines not only their cellular trafficking, but also their biophysical properties. They depend mainly on the presence or absence of the GluA2 subunit, which determines the Ca^2+^ permeability of AMPARs (Hollmann et al., 1991; Geiger et al., 1995). GluA2-containing AMPARs are impermeable to Ca^2+^ (calcium impermeable AMPARs, CI-AMPARs), whereas GluA2-lacking AMPARs are permeable to Ca^2+^ [calcium permeable AMPARs (CP-AMPARs)]. As mentioned above, recent studies in FXS animal models suggest that the loss of FMRP leads to numerous ion channel dysfunctions, including AMPARs. However, the obtained results are often inconsistent, showing both increased and decreased total or surface protein expression of GluA1 and/or GluA2 in *Fmr1* KO or FmrpR138Q mutant mice (Prieto et al., 2021). Moreover, the results in FXS models show brain region and cell-type-specific and age-dependent defects. Nevertheless, the overall picture of dysregulated AMPAR levels and/or properties in FXS is unclear. As mentioned previously, the E/I balance as well as the levels of ion channel receptors in FXS may differ across development; thus, different results obtained at different developmental stages could potentially be due to the drive to establish a new balance.

In the current study, we investigated the consequences of the loss of FMRP on the synaptic proteome in the adult, developed brains of *Fmr1* KO mice. Using quantitative high-resolution mass spectrometry, we analyzed the proteome of synapses (synaptoneurosomes, SN) isolated from adult *Fmr1* KO and WT mice. In *Fmr1* KO SN, we identified dysregulated levels of proteins involved in the regulation of glutamatergic synaptic transmission, postsynaptic receptor activity, and synapse organization. Furthermore, we report deficient synaptic accumulation of AMPARs in response to *in vitro* NMDAR stimulation in *Fmr1* KO SN. Finally, we demonstrate an increased steady-state surface level of GluA2-containing AMPARs in adult synapses of *Fmr1* KO mice in the synaptoneurosomal model, primary hippocampal neurons, and acute brain slices. In aggregate, in the present study, we show that *Fmr1* KO adult synapses display defective activity-induced AMPAR trafficking and enhanced steady-state surface levels of Ca^2+^-impermeable GluA2-containing AMPARs.

## Materials and methods

### Animals

In the study, 2- to 3-month-old male FVB mice (FVB/NJ, Jackson Laboratories Stock No.: 001800) were used. Before the experiment, the animals were kept in the laboratory animal facility under a 12-h light/dark cycle with food and water available *ad libitum*. The animals were treated in accordance with the EU Directive 2010/63/EU for animal experiments.

### Preparation of synaptoneurosomes and stimulation of NMDA receptors

Synaptoneurosomes were prepared as described previously (Scheetz et al., 2000; Dziembowska et al., 2012; Kuzniewska et al., 2018). Before tissue dissection, Krebs buffer (2.5 mM CaCl_2_, 1.18 mM KH_2_PO_4_, 118.5 mM NaCl, 24.9 mM NaHCO_3_, 1.18 mM MgSO_4_, 3.8 mM MgCl_2_, and 212.7 mM glucose) was aerated with an aquarium pump for 30 min at 4°C. Next, the pH was lowered to 7.4 using dry ice. The buffer was supplemented with 1 × protease inhibitor cocktail complete EDTA-free (Roche). Animals were euthanized by cervical dislocation; hippocampi and a part of the cortex adjacent to the hippocampus (containing the subiculum, entorhinal, perirhinal, postrhinal, and visual cortex) were dissected. The tissue from one hemisphere (~50 mg) was homogenized in 1.5 mL of Krebs buffer using the Dounce homogenizer with 10–12 strokes. All steps were kept ice-cold to prevent the stimulation of synaptoneurosomes. Homogenates were loaded into a 20-mL syringe and passed through a series of pre-soaked (with Krebs buffer) nylon mesh filters of 100, 60, 30, and 10 μm (Merck Millipore) in a cold room to a 50-mL polypropylene tube, centrifuged at 1,000 *g* for 15 min at 4°C, washed, and the pellet was resuspended in Krebs buffer with protease inhibitors. The protocol for the *in vitro* stimulation of NMDA receptors on synaptoneurosomes was described before (Scheetz et al., 2000; Kuzniewska et al., 2018). Briefly, the aliquots of freshly isolated synaptoneurosomes were prewarmed for 3 min at 37°C and stimulated with a pulse of 50 μM NMDA and 10 μM glutamate for 30 s, then APV (120 μM) was added, and synaptoneurosomes were further incubated for the indicated time (1–5 min) at 37°C. Unstimulated samples kept on ice were used as controls.

### Western blot analysis of synaptoneurosome preparations

Equal amounts of protein from the homogenate and synaptoneurosomal fractions were resolved on SDS-PAGE (10%, TGX Stain-Free FastCast Acrylamide Solutions, Bio-Rad). Proteins were transferred to PVDF membranes (pore size 0.45 μm, Immobilon-P, Merck Millipore) using the Trans-Blot Turbo Blotting System (Bio-Rad; 170-4155). Membranes were blocked for 1 h at room temperature in 5% non-fat dry milk in PBS-T (PBS with 0.01% Tween-20), followed by overnight incubation at 4°C with primary antibodies (PSD95 Cat#MAB1598, Merck Millipore; Nlgn1 Cat#129111, Synaptic Systems; synaptophysin Cat#MAB329, Merck Millipore; Gapdh Cat#MAB374, Merck Millipore; c-Jun Cell Signaling Cat#9165, KDM1/LSD1 Cat#ab129195, Abcam) in 5% milk in PBS-T. Blots were washed 3 × 5 min with PBS-T, incubated 1 h at room temperature with HRP-conjugated secondary antibody (1:10,000 in 5% milk), and washed 3 × 5 min with PBS-T. The HRP signal was detected using the Amersham ECL Prime Western Blotting Detection Reagent (GE Healthcare) on the Amersham Imager 600 using automatic detection settings.

### Proteomics

Synaptoneurosomes were isolated from P70 (postnatal day 70) male WT and *Fmr1* KO mice (*n* = 4 per genotype), and pellets were snap-frozen at −80°C directly after the isolation. Next, samples were dissolved in neat trifluoroacetic acid (TFA). Protein solutions were neutralized with 10 volumes of 2M Tris base, supplemented with TCEP (8 mM) and chloroacetamide (32 mM), heated to 95°C for 5 min, diluted with water in a ratio of 1:5, and subjected to overnight enzymatic digestion with sequencing-grade modified trypsin (Promega) at 37°C (Doellinger et al., 2020). Tryptic peptides were then desalted on C18 stage tips, TMT-labeled on the solid support (Myers et al., 2019), compiled into a single TMT sample, and then concentrated. Peptides in the compiled sample were fractionated into eight fractions using a high-pH reversed-phase peptide fractionation kit (Thermo Fisher Scientific) and concentrated. Prior to LC-MS measurement, the peptide fractions were resuspended in 0.1% TFA and 2% acetonitrile in water. Chromatographic separation was performed on an Easy-Spray Acclaim PepMap column with 15 cm long × 75 μm inner diameter (Thermo Fisher Scientific) at 35°C by applying 105 min of acetonitrile gradients in 0.1% aqueous formic acid at a flow rate of 300 nl/min. An UltiMate 3000 nano-LC system was coupled with a Q Exactive HF-X mass spectrometer *via* an easy-spray source (all Thermo Fisher Scientific). The Q Exactive HF-X was operated in TMT mode with survey scans acquired at a resolution of 60,000 at m/z 200. Up to 15 most abundant isotope patterns with charges 2–5 from the survey scan were selected with an isolation window of 0.7 m/z and fragmented by higher-energy collision dissociation (HCD) with normalized collision energies of 32, while the dynamic exclusion was set to 35 s. The maximum ion injection times for the survey scan and the MS/MS scans (acquired with a resolution of 45,000 at m/z 200) were 50 and 96 ms, respectively. The ion target value for MS was set to 3e6 and for MS/MS to 1e5, and the minimum AGC target was set to 1e3. The data were processed with MaxQuant v. 1.6.17.0 (Tyanova et al., 2016a), and the peptides were identified from the MS/MS spectra searched against the UniProt Mouse Reference Proteome (UP000000589) using the built-in Andromeda search engine. Reporter ion MS2-based quantification was applied with reporter mass tolerance = 0.003 Da and min. reporter PIF = 0.75. Cysteine carbamidomethylation was set as a fixed modification, and methionine oxidation, glutamine/asparagine deamination, and protein N-terminal acetylation were set as variable modifications. For *in silico* digests of the reference proteome, cleavages of arginine or lysine followed by any amino acid were allowed (trypsin/P), and up to two missed cleavages were allowed. The FDR was set to 0.01 for peptides, proteins, and sites. A match between runs was enabled. Other parameters were used as pre-sets in the software. Reporter intensity-corrected values for protein groups were loaded into Perseus v. 1.6.10.0 (Tyanova et al., 2016b). Standard filtering steps were applied to clean up the dataset: reverse (matched to the decoy database), only identified by site, and potential contaminant (from a list of commonly occurring contaminants included in MaxQuant) protein groups were removed. Reporter intensity corrected values were log2 transformed, and protein groups with all values were kept. Reporter intensity values were then normalized by median subtraction within TMT channels. Student *t*-testing was performed on the dataset to return protein groups, in which levels were statistically and significantly changed between sample groups (*p*-value < 0.05). Gene annotation enrichment analysis was performed using DAVID (https://david.ncifcrf.gov/tools.jsp).

The mass spectrometry proteomics data have been deposited with the ProteomeXchange Consortium *via* the PRIDE partner repository with the dataset identifier PXD043700. Reviewer account details: Username: reviewer_pxd043700@ebi.ac.uk; Password: HXS1vVmT.

### BS3-crosslinking of surface-expressed proteins

The aliquots of WT and *Fmr1* KO synaptoneurosomes after NMDAR stimulation were either rapidly frozen on dry ice (total protein levels) or incubated with the cell membrane-impermeable BS3 crosslinker [BS3 (bis(sulfosuccinimidyl)suberate], Thermo Fisher Scientific), as described previously (Boudreau et al., 2012). Briefly, BS3 crosslinker was prepared as a 52-mM stock solution in a 5-mM sodium citrate buffer, pH = 5. SNs were incubated with BS3 crosslinker at a final concentration of 2 mM for 30 min at 4°C. Then, 100 mM glycine was added and incubated for 10 min at 4°C to quench the remaining unbound BS3. Next, the samples were rapidly frozen on dry ice and stored at −80°C.

### Western blot analysis of surface, intracellular, and total GluA1, GluA2, and GluA3 protein levels

Total (BS3 untreated) and BS3-crosslinked samples were diluted with Laemmli loading buffer and denatured at 96°C. Proteins were resolved on SDS-PAGE (10%, TGX Stain-Free FastCast Acrylamide Solutions, Bio-Rad), and equal protein loading was verified using the Gel Doc XR+ Gel Documentation System (Bio-Rad). Proteins were transferred to PVDF membranes (pore size 0.45 μm, Immobilon-P, Merck Millipore) using a Trans-Blot SD semi-dry transfer cell (Bio-Rad) for 1.5 h. Membranes were blocked at room temperature for 1.5 h in 5% BSA and 5% NGS in PBS-T (PBS with 0.01% Tween-20), followed by overnight incubation with primary antibody (1:1,000 in 5% BSA) at 4°C. The crosslinked and non-crosslinked samples were probed with antibodies for different AMPAR subunits: anti-GluR1 (Thermo Scientific, Pierce, cat. # PA1-37776), anti-GluR2 (Cell Signaling, cat. # 13607), and anti-GluR3 (Cell Signaling, cat. # 3437). Blots were washed for 4 × 15 min with PBS-T and incubated for 1 h at room temperature with HRP-conjugated secondary antibody (1:10,000 in 3% BSA) and washed for 4 × 15 min in PBS-T. The HRP signal was detected using the Amersham ECL Prime Western Blotting Detection Reagent (GE Healthcare) on the Amersham Imager 600 (GE Healthcare).

### Electrophysiology

In the study, 1.5-month-old male mice were used for electrophysiological recordings. To obtain acute brain slices, mice were anesthetized with isoflurane and decapitated. Coronal brain slices (250 μm thick) were prepared using Leica VT 1200S vibratome in ice-cold NMDG cutting solution (135 mM NMDG, 1 mM KCl, 1.2 mM KH_2_PO_4_, 1.5 mM MgCl_2_, 0.5 mM CaCl_2_, 20 mM choline bicarbonate, and 10 mM D-glucose, bubbled with carbogen (5% CO_2_, 95% O_2_). Slices containing the hippocampus were collected and transferred to a beaker filled with ACSF solution [119 mM NaCl, 2.5 mM KCl, 1 mM NaH_2_PO_4_, 26 mM NaHCO_3_, 1.3 mM MgCl_2_, 2.5 mM CaCl_2_, and 10 mM D-glucose, bubbled with carbogen (5%CO_2_, 95% O_2_)] and incubated for 12–15 min at 34°C. Then, the beaker containing slices was placed on the bench at room temperature, where they remained for the rest of the experiment. Electrophysiological recordings began at least 1 h after the slicing procedure. Slices were transferred to the recording chamber, perfused with the ACSF solution supplemented with 50 μM picrotoxin, heated to 31°C, and constantly bubbled with carbogen. Hippocampal CA1 neurons were identified visually and patched with a borosilicate glass pipette of 4–6 MΩ resistance, and filled with the cesium-based internal solution: 130 mM Cs gluconate, 20 mM HEPES, 3 mM TEA-Cl, 0.4 mM EGTA, 4 mM Na_2_ATP, 0.3 mM NaGTP, 4 mM QX-314Cl, pH = 7.0, and osmolarity: 292 mOsm. A stimulating, bipolar electrode filled with ACSF solution was placed in the Schaffer collaterals. To trigger synaptic release, the electrode generates pulses every 5 s. Series and input resistances were monitored throughout the recording. Peak amplitudes of AMPA receptor-mediated EPSCs were measured for 5–15 min. After that time, ACSF supplemented with 100 μM 1-naphthylacetyl spermine trihydrochloride (NASPM) was perfused through the chamber, while the recording continued for another 15 min. The amplitude of AMPAR EPSCs before NASPM application was calculated by averaging a 3-min epoch of baseline right before NASPM perfusion. The NASPM effect was shown by averaging a 3-min epoch recorded after a minimum of 10 min after NASPM application. A total number of recorded cells: WT *n*_cells_ = 9 from 2 animals, KO *n*_cells_ = 8 from 2 animals.

### Primary mouse hippocampal cultures

Dissociated hippocampal cultures were prepared from postnatal day 0 (P0) *Fmr1* KO and WT male mice as described previously with minor modifications (Moutin et al., 2020). The hippocampi were dissected and transferred into a falcon tube with ice-cold Hibernate A (Thermo Fisher Scientific) and penicillin/streptomycin (P/S, Sigma Aldrich, 1%). Then the enzyme solution [Hibernate, Papain 200–250U (Worthington)] was added, and the hippocampi were incubated for 15–20 min at 37°C. Next, the hippocampi were washed with the FBS (fetal bovine serum) medium DMEM (Thermo Fisher Scientific), FBS 10% (Thermo Fisher Scientific), and P/S 1%. After washing, the tissue was triturated in a small amount of media and shortly centrifuged for 7 min at 300 *g* at room temperature. Next, the necessary amount of FBS medium was added, and the cells were plated (100 ul/well in a 12-well plate) in a droplet. After incubation of the cells at 37° and 5% CO_2_ for 60 min, 1 mL of NBA medium was added (Neurobasal A, Thermo Fisher Scientific; B27 2%, Gibco; GlutaMAX supplement 2 mM, Gibco, Cat#35050038; P/S 1%). For immunostaining and GluA2 content analysis, the cells were plated at a density of 90,000 cells per 18-mm-diameter coverslip (Assistant, Germany) coated with 50 μg/mL of poly-D-lysine (Sigma Aldrich, Cat# P8920). The cultures were kept at 37°C in 5% CO_2_ in a humidified incubator. The experiments were performed on the 19th day *in vitro* (DIV).

### Surface staining of GluA2 receptors

Surface staining of neurons was performed as previously described (Lu et al., 2001; Lee et al., 2004) with minor modifications. In total, 19 DIV neurons were fixed under non-permeabilizing conditions by incubation in 4% paraformaldehyde/4% sucrose in PBS for 5 min at room temperature and washed three times in PBS for 10 min. Fixed cells were incubated in the blocking solution (5% normal goat serum in PBS) for 2 h/RT. Next, the neurons were incubated with primary anti-GluA2 antibody (Merck Millipore, cat. #MAB397) 1:200 in the blocking solution overnight at 4°C to label surface receptors. After washing (3x PBS, 10 min), antibody-labeled surface receptors were stained with Alexa488-conjugated secondary antibody (Life Technologies, #A11001) for 1 h at RT. After washing, the cells were mounted in Fluoromount-G (Invitrogen, cat# 00-4958-02).

### Imaging and quantification of surface GluA2 protein

Images of stained secondary and tertiary dendrites were acquired under 488 nm fluorescent illumination using the Zeiss LSM700 confocal microscope (63x objective, 1.4 NA) at a pixel resolution of 1,024 × 1,024 with a 1.4 zoom, resulting in a 0.07 μm pixel size. For picture analysis, ImageJ software was used. Z-stacks were combined into one maximum-intensity projection. Dendrites were analyzed separately using regions of interest (ROI). A high threshold was set within each ROI to create a mask that segmented the dendrite as an area for fluorescence measurement. The mean fluorescence intensity for every mask within the ROI was used. Four independent cell cultures per genotype were used for analysis, with five images from each culture. The number of examined ROIs for the wild type was *n* = 102, and for *Fmr1* KO, *n* = 66.

### Quantification and statistical analysis

Unless otherwise noted, statistical analysis was performed using GraphPad Prism 9.3 (GraphPad Software, Inc.). Statistical details of experiments, including the statistical tests used and the value of n, are noted in the figure legends.

## Results

### Synaptic proteome of adult *Fmr1* KO mice

**Figure 1:**
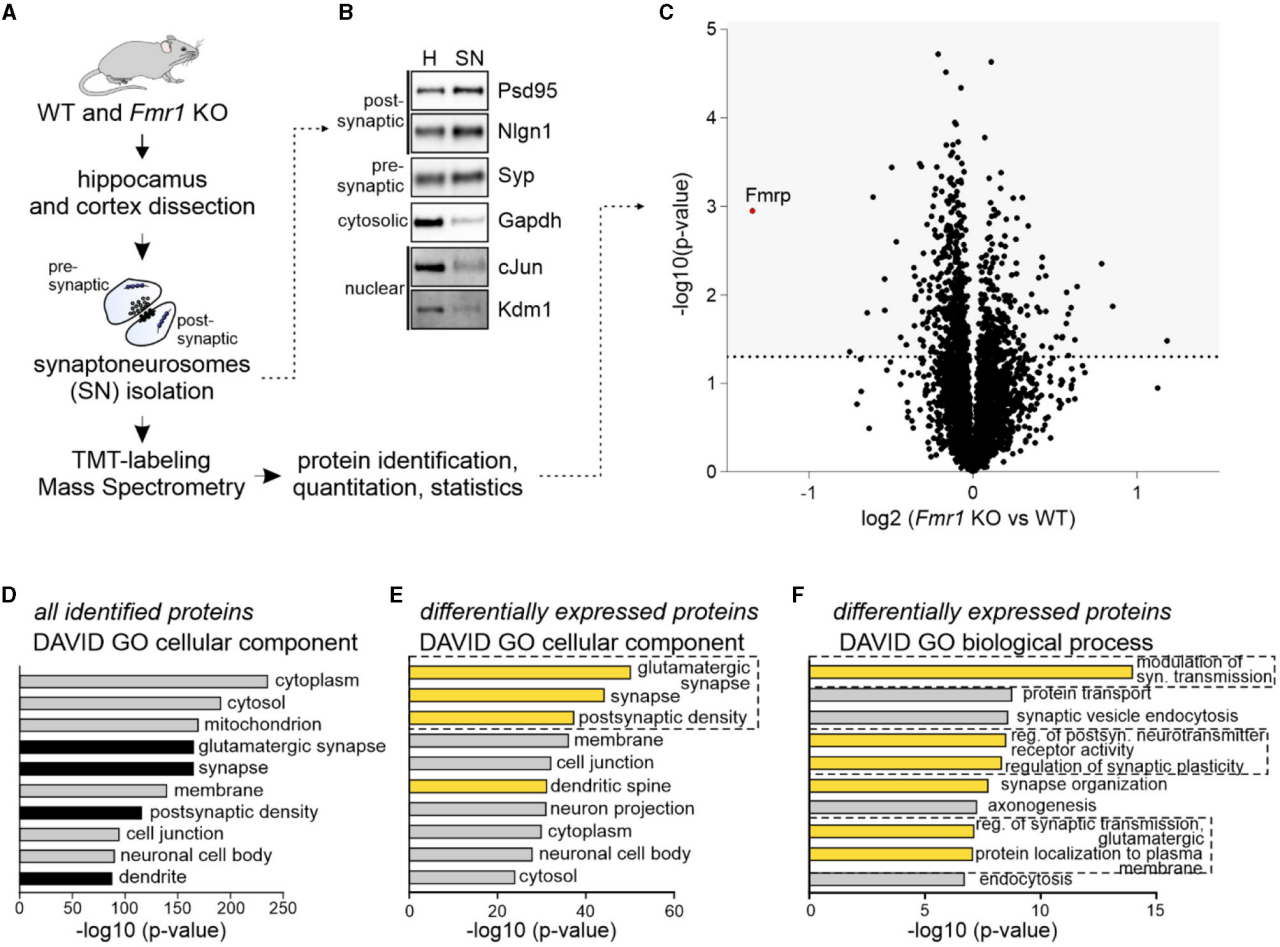
Quantitative mass spectrometry analysis of synaptoneurosomes from adult *Fmr1* KO mice. **(A)** Schematic illustration of the experimental workflow depicting synaptoneurosomes (SNs) isolation and proteomics. **(B)** Western blot on fractions obtained during SN preparation reveals the enrichment of both pre- and postsynaptic markers in the SN fraction. Cytosolic and nuclear markers were depleted in the SN. **(C–F)** Results of high-resolution quantitative mass spectrometry. **(C)** Volcano plot showing an abundance of identified proteins in *Fmr1* KO SNs as compared to WT SNs. The vertical line defines the p-value statistical significance cutoff (-log10 *p*-value > 1.3; Student *t*-test, *n* = 4 per group). **(D, E)** DAVID gene ontology analysis of “cellular component” annotation of proteins. **(D)** In synaptoneurosomal samples, the top categories were cytoplasmic, mitochondrial, synaptic, and membrane proteins. **(E, F)** DAVID analysis of differentially expressed proteins in *Fmr1* KO SNs showed the top three “cellular component” categories as glutamatergic synapse, synapse, and postsynaptic density. Among the top “biological process” categories proteins involved in the modulation of glutamatergic synaptic transmission, the regulation of postsynaptic neurotransmitter receptor activity, synapse organization, and protein localization to the plasma membrane were identified.

To investigate the synaptic proteome of adult *Fmr1* KO mice, we used the quantitative mass spectrometry method based on isobaric labeling and synaptoneurosomes (SNs), preparations freshly obtained from the brain and enriched in synapses, containing both pre- and postsynaptic compartments. We isolated SNs from the hippocampus and somatosensory cortex of *Fmr1* KO and WT littermates (P70) (Figure 1A). The Western blot on fractions obtained during SN preparation revealed the enrichment of both pre- and postsynaptic markers and the depletion of cytosolic and nuclear markers in the SN fraction as compared to the homogenate (Figure 1B). SN samples were labeled with TMT tags (four replicates per genotype) before MS. We identified 4,931 proteins primarily with cytoplasmic, mitochondrial, and synaptic localization, as shown by DAVID gene annotation analysis (Figures 1C, D). In total, 776 proteins were significantly up or downregulated in *Fmr1* KO SNs, with the most significantly downregulated being FMRP itself (Figure 1C). The list of identified proteins is attached as Supplementary Table 1. DAVID GO “cellular component” analysis performed on the group of proteins significantly dysregulated in *Fmr1* KO samples revealed glutamatergic synapse and postsynaptic density proteins as the most abundant ones (Figure 1E). To gain more insight into the molecular functions of identified up and downregulated proteins, we performed DAVID GO “biological process” analysis, which revealed the overrepresentation of proteins involved in the modulation of glutamatergic synaptic transmission, regulation of postsynaptic receptor activity, synapse organization, and protein localization to the plasma membrane (Figure 1F).

### Activity-induced shuttling of AMPA receptors in *Fmr1* KO synapses

**Figure 2:**
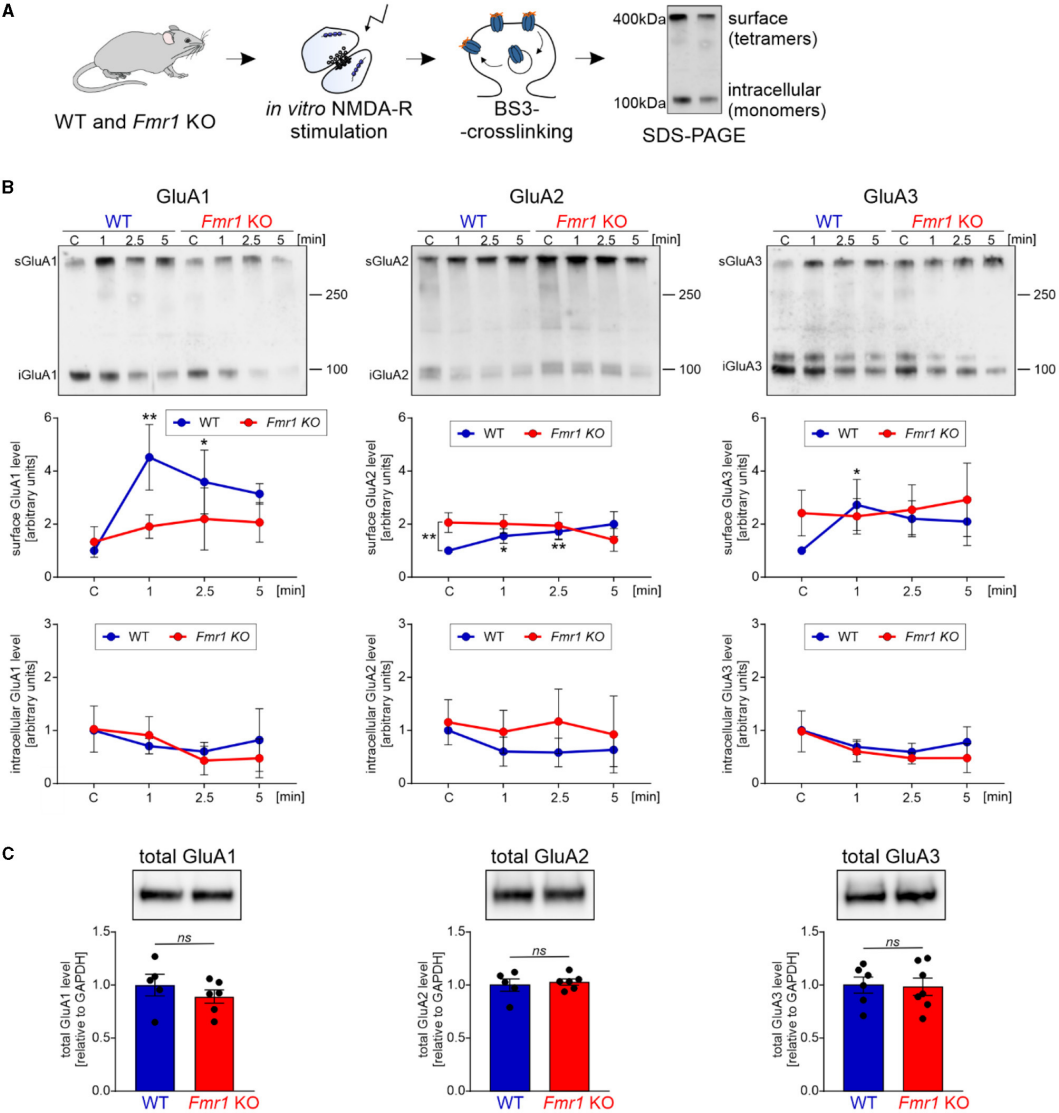
*Fmr1* KO synaptoneurosomes display increased surface levels of GluA2 at the basal state and do not increase/accumulate surface AMPAR in response to stimulation. **(A)** Schematic illustration of the experimental workflow depicting synaptoneurosomes (SNs) isolation and NMDAR *in vitro* stimulation, followed by surface protein crosslinking, SDS-PAGE, and Western blotting. **(B)** WT and *Fmr1* KO synaptoneurosomes at the basal state (control, C, basal state) and stimulated for 1, 2.5, and 5 min were subjected to surface protein crosslinking. Representative immunoblots show surface AMPARs (tetramers) at ~400 kDa and intracellular AMPARs (monomers) at ~100 kDa. Band intensities were calculated according to WT C, which was set as “1”. Data are presented as mean ± SEM. In the WT synaptoneurosomes after the stimulation, a rapid increase in the surface levels of AMPAR was observed (GluA1: *n* = 3; 1′ vs. C **p = 0.0066; 2.5′ vs. C *p = 0.0278; GluA2: *n* = 3; 1′ vs. C *p = 0.0423; 2.5′ vs. C **p = 0.0097; GluA3: *n* = 4; 1′ vs. C *p = 0.0358; repeated measures two-way ANOVA, *post-hoc* Tukey's multiple comparisons test). In contrast, in *Fmr1* KO samples, SN stimulation did not influence AMPAR surface levels at any analyzed timepoints (RM two-way ANOVA, *post-hoc* Tukey's multiple comparisons test, *p* > 0.05). Interestingly, we observed increased levels of GluA2-containing AMPARs at the basal state in the *Fmr1* KO SNs (GluA2: *n* = 3; WT C vs. KO C **p = 0.0074; RM two-way ANOVA, *post-hoc* Sidak's multiple comparisons test) (see Supplementary Figure S1). **(C)** Analysis of total GluA1, GluA2, and GluA3 protein levels in WT and *Fmr1* KO SNs did not reveal any significant changes in AMPAR subunits among the two genotypes (WT, *n* = 5; Fmr1 KO, *n* = 6; unpaired *t*-test; *p* > 0.05).

The majority of fast excitatory synaptic transmission in the brain is mediated by the AMPA receptors localized at the postsynaptic density. We asked whether the observed dysregulation of proteins involved in synapse organization and protein localization to the plasma membrane in *Fmr1* KO mice may result in altered transport of AMPA receptors and their subunit composition. Synaptic AMPA receptor trafficking is a dynamic process controlled by neuronal activity. AMPA receptors shuttle between the cell surface and intracellular compartments, whereas NMDA receptor proteins are relatively fixed (Bredt and Nicoll, 2003). To look at AMPA receptor levels in the synapse at a particular moment, we needed to distinguish between the surface and intracellular pools of AMPAR. For this, we used the surface protein crosslinking method with the BS3-membrane-impermeable reagent, followed by SDS-PAGE and Western blotting with specific antibodies for GluA1, GluA2, and GluA3 subunits. This method allows us to distinguish between the synaptic, membrane-bound AMPAR crosslinked tetramers (bands of ~400 kDa) and intracellular AMPARs that occur on the SDS-PAGE gel as ~100 kDa monomers (Figure 2A). To study the dynamics of AMPAR shuttling, we stimulated SNs isolated from *Fmr1* KO and WT mice brains for 1, 2.5, and 5 min with the NMDAR stimulation protocol described before (Scheetz et al., 2000; Kuzniewska et al., 2018). Next, the surface and intracellular pools of AMPARs were analyzed. As early as 1 min after the NMDAR stimulation, we observed increased surface localization of GluA1 and, to a lesser extent, also GluA2 and GluA3 in SNs isolated from WT mice brains (Figure 2B, upper graphs, WT—blue lines; ^*^p < 0.05, ^**^p < 0.01). In contrast, in *Fmr1* KO SNs, the stimulation of NMDAR did not induce a synaptic incorporation of AMPAR subunits at any of the analyzed timepoints (Figure 2B, upper graphs, *Fmr1* KO—red lines; *ns p* > 0.05). Interestingly, when we compared the basal level of AMPA receptor subunits in non-stimulated WT and *Fmr1* KO SNs, we discovered increased surface levels of GluA2 protein in *Fmr1* KO mice (Figure 2B, upper, middle graph, ^**^p < 0.01). Total protein levels of GluA1, GluA2, or GluA3 subunits of AMPARs did not differ in *Fmr1* KO and WT SNs in the standard Western blot analysis (Figure 2C, *ns p* > 0.05).

**Figure 3:**
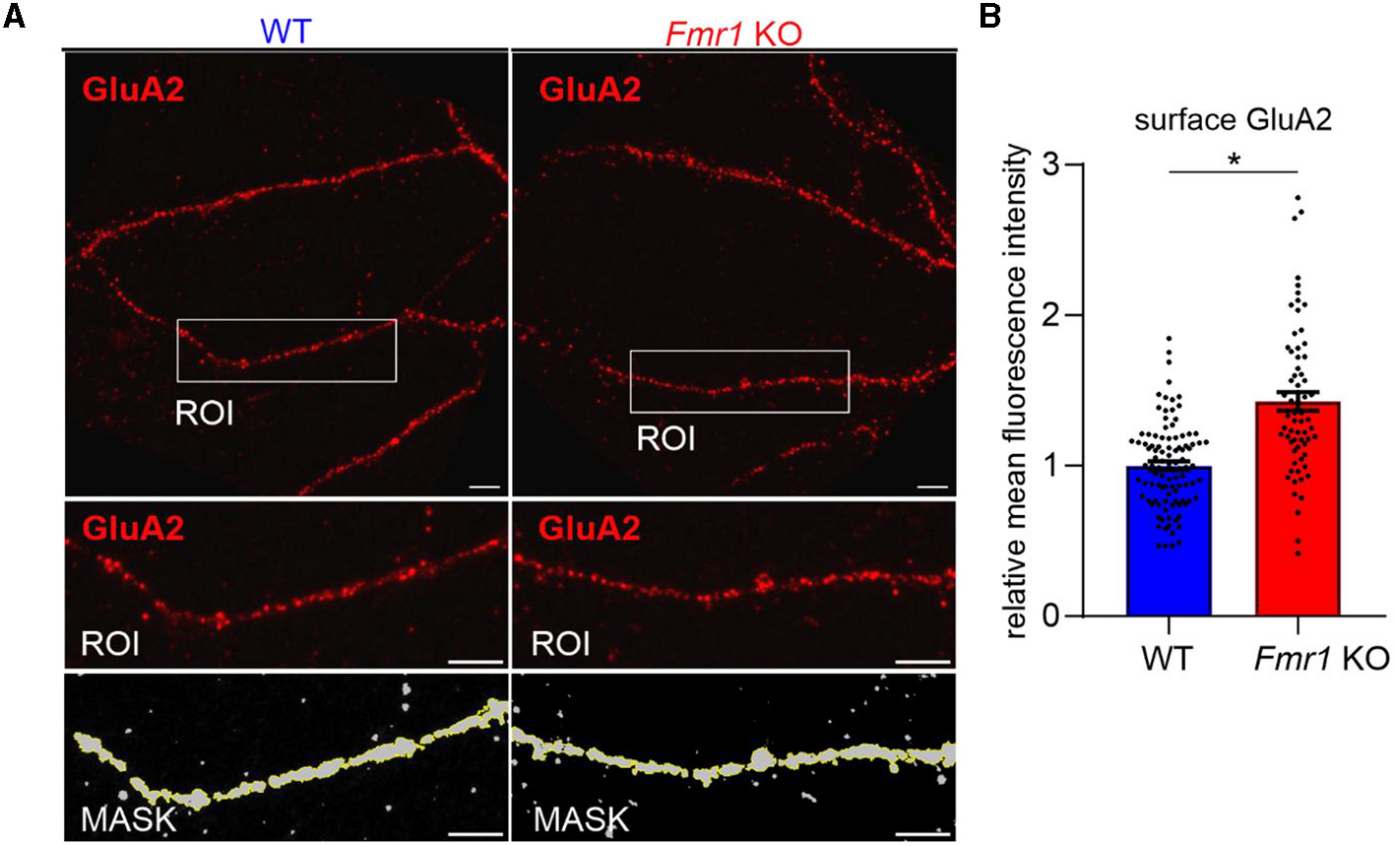
Increased levels of surface GluA2 in hippocampal neurons of *Fmr1* KO mice. **(A)** Upper: Representative immunofluorescence images of secondary dendrites from WT and *Fmr1* KO hippocampal neurons at 19 DIV stained for surface GluA2. Scale bar: 5 μm. Lower: Magnified images of boxed areas. A thresholding mask was created to segment the target dendrite, and the mean fluorescent intensity was measured within the mask. **(B)** The graph shows the results of the quantification of the mean surface GluA2 fluorescent intensity. Values were relativized to the average GluA2 intensity in WT, and data are presented as mean ± SEM (*p = 0.015; nested *t*-test; *n* = 66–102 ROIs analyzed/genotype, *N* = 4 independent neuronal cultures/genotype).

As we observed increased surface levels of GluA2 protein in *Fmr1* KO synaptoneurosomes at basal, non-stimulated conditions, we aimed to assess surface GluA2 levels in another experimental model. We performed immunolabeling with the anti-GluA2 antibody on cultured primary hippocampal neurons (19 DIV) from WT and *Fmr1* KO mice. The immunostaining was performed in non-permeabilized conditions to visualize only surface receptors. After imaging with a confocal microscope, we observed increased surface levels of the GluA2 subunit of AMPAR in the dendrites of *Fmr1* KO neurons as compared to WT (Figure 3, *p* < 0.05).

### Electrophysiological evaluation of the abundance of Ca^2+^-impermeable and Ca^2+^-permeable AMPA receptors at the synapses of *Fmr1* KO mice

GluA2 has an especially important role because, following post-transcriptional editing at the Q/R site at position 607, the AMPAR channels become Ca^2+^-impermeable. As in the adult brain, the vast majority of GluA2 subunits are in the edited form (Carlson et al., 2000), the presence of the GluA2 subunit in the AMPA receptor determines its Ca^2+^-permeability.

**Figure 4:**
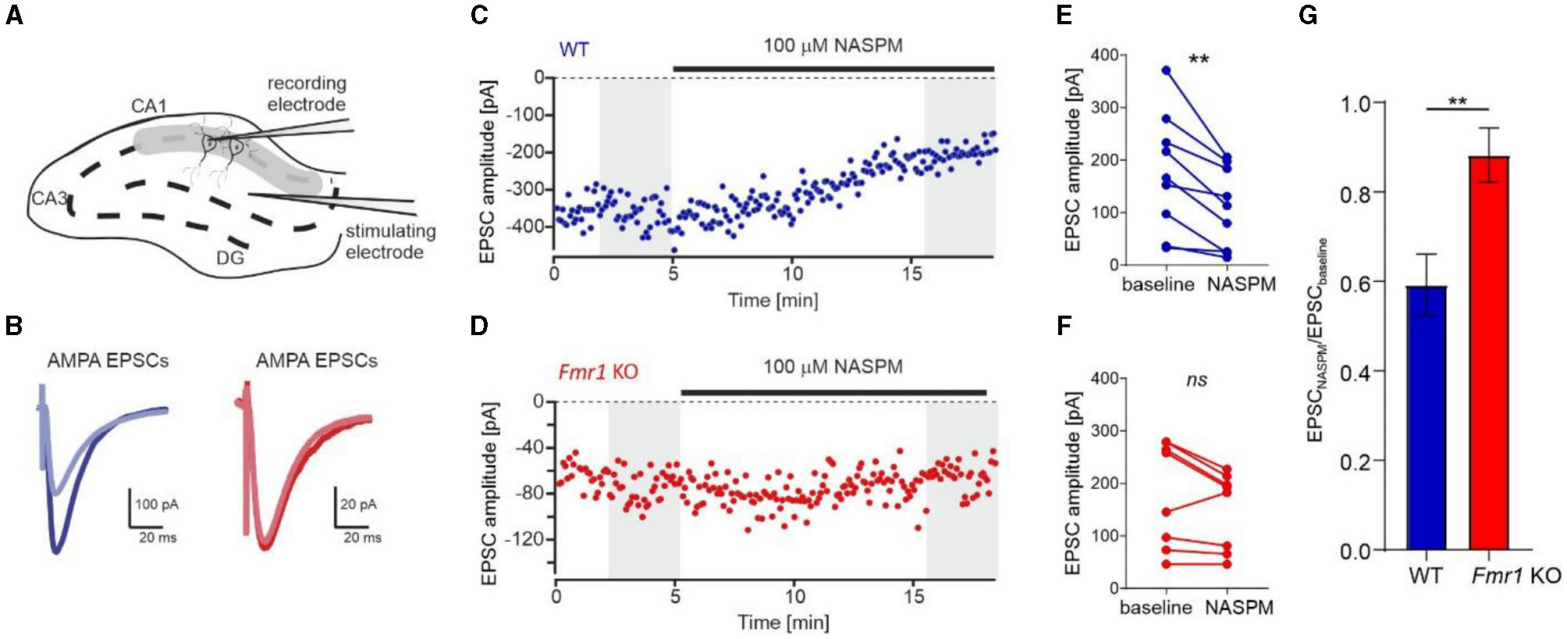
Increased levels of Ca^2+^-impermeable AMPAR *(GluA2-containing AMPAR)* at the synapses of *Fmr1* KO mice. **(A)** Diagram showing the positions of the stimulating and recording electrodes in the CA1 field of the hippocampus. **(B)** Example AMPAR-mediated EPSCs averaged from the first 60 traces (navy blue, WT, or dark red, *Fmr1* KO) and the last 60 traces (light blue or light red) of the recordings shown in **(C, D)**. **(C, D)** Representative recordings of AMPA receptor-mediated EPSCs of CA1 neurons from WT (blue) and *Fmr1* KO (red) illustrate the effect of NASPM after 10 min of bath application. **(E, F)** Averaged amplitudes of each recorded cell before and after the application of NASPM (grayed areas from **C, D** panels) showing a decrease of EPSCs amplitude following bath application of 100 μM NASPM in WT (**p = 0.0034, paired *t*-test) but not *Fmr1* KO (ns, *p* = 0.067, paired *t*-test). **(G)** Bar graph summarizing NASPM-induced decrease of AMPARs EPSCs amplitudes, suggesting higher abundance of GluA2 subunits in the CA1 of *Fmr1* KO mice (unpaired *t*-test, **p = 0.0069).

To support our data and prove conclusively that an increased number of GluA2-containing AMPARs is incorporated into *Fmr1* KO synapses, we performed electrophysiological recordings on acute brain slices from adult *Fmr1* KO and WT mice. We used 1-naphthylacetyl spermine trihydrochloride (NASPM), a selective antagonist of Ca^2+^-permeable AMPARs, to block GluA2-lacking AMPAR and measured AMPAR-mediated EPSCs *via* whole-cell voltage-clamp recording in the CA1 of acute brain hippocampal slices (Figure 4A). The bath application of NASPM significantly decreased EPSC amplitude in neurons recorded from WT slices, thus blocking the contribution of GluA2-lacking AMPARs in recorded current amplitudes (Figures 4B, C, E, ^**^p = 0.0034, paired *t*-test). In contrast, the application of NASPM had no significant effect on EPSC amplitude in *Fmr1* KO neurons (Figures 4B, D, F, *ns, p* = 0.067, paired *t*-test). We compared the effect of NASPM, illustrated as the AMPARs EPSCs amplitude normalized to baseline, in the two genotypes and found an increased ratio of EPSC amplitudes in *Fmr1* KO cells (Figure 4G, ^**^p = 0.0069, unpaired *t*-test). Altogether, the obtained data corroborate the idea of increased levels of GluA2-containing AMPARs at the synapses of adult *Fmr1* KO mice.

## Discussion

Using a variety of complementary experimental strategies, the present study provides evidence for impaired activity-dependent synaptic delivery of AMPARs in adult *Fmr1* KO synapses. Moreover, we report a switch in the synaptic AMPAR subtype toward an increased number of GluA2-containing Ca^2+−^impermeable receptors in adult *Fmr1* KO synapses.

In the present study, we used high-resolution quantitative mass spectrometry to compare the synaptic proteome of adult *Fmr1* KO and their littermate wild-type mice. We detected changes in the abundance of proteins regulating glutamatergic synapses and postsynaptic receptor activity. The dynamic shuttling of AMPA receptors is the main mechanism that regulates their membrane abundance. We used NMDAR stimulation to induce AMPAR trafficking, followed by their crosslinking at the surface. Our study revealed that *Fmr1* KO SNs do not respond to stimulation with fast AMPAR membrane incorporation as observed for wild types. Next, using biochemical and electrophysiological methods, we demonstrated an increased level of GluA2-containing AMPA receptors in the synapses of *Fmr1* KO mice.

Numerous studies focused on the analysis of the transcriptome and proteome of *Fmr1* KO mouse brains, but the resulting picture is still incoherent (Brown et al., 2001; Darnell et al., 2011; Ascano et al., 2012; Tang et al., 2015; Das Sharma et al., 2019). The discrepancies in protein enrichment observed by the authors are likely the result of different brain region/cell type usage and, most importantly, the developmental stages of animals. Previous proteomic studies have shown that the impact of FMRP protein deletion on the synaptic proteome depends on the age of animals (Tang et al., 2015). While a large number of proteins were upregulated in very young, 17-day-old *Fmr1* KO mice, these differences were largely absent in adulthood (Tang et al., 2015). Nevertheless, although the altered proteome of *Fmr1* KO mice stabilizes with age, the development of neuronal circuits and synaptic connections is influenced by the most changed proteome. A population of proteins that remains dysregulated in adulthood can affect the physiological properties of *Fmr1* KO synapses; therefore, we aimed at their characterization with quantitative proteomics. Consistent with the previous data, we did not observe massive alterations in the proteome of synaptoneurosomes isolated from adult *Fmr1* KO mice. Nevertheless, we did observe changes in several groups of proteins, such as those involved in the modulation of glutamatergic synaptic transmission, synapse organization, and protein localization to the plasma membrane. While we identified “regulation of postsynaptic receptor activity” as one of the major categories among the dysregulated proteins, we found no changes in the AMPAR subunits themselves. This fact encouraged us to study the synaptic level of AMPA receptors and the dynamics of their synaptic distribution.

AMPARs mediate the majority of fast excitatory synaptic transmission in the brain. Four subunits, GluA1–GluA4, form tetramers, and different combinations possess unique biophysical properties and trafficking behavior (Shepherd and Huganir, 2007). We used the BS3-crosslinking method to measure the surface and intracellular levels of GluA1, GluA2, and GluA3 subunits in *Fmr1* KO and WT synapses in response to NMDAR stimulation. We show that the *in vitro* stimulation of wild-type SNs leads to the dynamic incorporation of AMPA receptor subunits GluA1, GluA2, and GluA3 into the synaptic membrane. However, this is not true for *Fmr1* KO, which does not increase the number of AMPARs on synapses in response to stimulation.

Discrepancies on whether GluA1 and/or GluA2 levels are affected in *Fmr1* KO mice exist in the literature. GluA1 protein levels have been shown to be reduced in the cortex but not in the hippocampus of *Fmr1* KO mice (Li et al., 2002). In another study, Nakamoto et al. (2007) reported decreased surface GluA1 protein levels in primary hippocampal neurons transfected with siRNA targeting FMRP. Next, Hu et al. (2008) used electrophysiological recordings of cultured hippocampal and cortical slices transfected with GFP-tagged GluA1 and GluA2 constructs. By measuring AMPA-mediated responses, they concluded that in *Fmr1* KO neurons, the synaptic delivery of GluA1 is impaired. More recently, Guo et al. (2015) have shown that in primary hippocampal neurons and in the hippocampi of *Fmr1* KO mice, the total GluA1 levels are comparable, but the membrane GluA1 levels are significantly reduced. In our study, we did not detect a decrease in the surface GluA1 level under basal conditions, and this discrepancy may be due to different experimental models. Altogether, our data show that the absence of FMRP leads to impaired activity-dependent synaptic shuttling of AMPA receptor subunits of GluA1, GluA2, and GluA3, without impairing overall GluA1-3 protein levels.

Furthermore, in our study, we discovered a specific increase in the abundance of the surface GluA2 subunit in *Fmr1* KO. Recently, increased *Gria2* expression at both the mRNA and protein levels was reported in the dendrites of hippocampal *Fmr1* KO neurons (14–17 DIV). In the same study, an increased level of functional GluA2-containing AMPARs was reported in the CA1 interneurons of juvenile (P14–P21) *Fmr1* KO mice (Hwang et al., 2022). Interestingly, another group observed a transient increase in GluA2-containing AMPARs at P6–P9 in *Fmr1* KO mice (Banke and Barria, 2020). In contrast to the abovementioned results, a recent study in neural progenitor cells from humans with FXS and *Fmr1* KO mice showed decreased *Gria2* and *Gria1* mRNAs and an increased number of GluA2-lacking AMPAR (Achuta et al., 2018). Some of the described discrepancies can be explained, at least in part, by differences in the developmental stages or experimental systems that included cultured hippocampal neurons, brain slices and homogenates, synaptoneurosomes, or neural progenitors induced from pluripotent stem cells. It is worth mentioning that AMPAR subunits GluR2–4 undergo RNA editing at the R/G site that differs depending on the developmental stage. R/G editing increases during neural development, resulting in faster desensitization and faster recovery rates of edited receptors (Lomeli et al., 1994). In this way, reduced editing at the R/G site can compensate for glutamate overstimulation. GluA2 subunits are also edited at the Q/R site, which changes channel properties toward Ca^2+−^impermeability. GluA2 is rapidly edited at the Q/R site during neural differentiation (Pachernegg et al., 2015), and in the adult human brain, as well as in mice, practically all GluA2 subunits are edited. Thus, GluA2-containing AMPARs are Ca^2+−^impermeable.

Numerous reports point toward the transient upregulation of a number of proteins in FXS, including GluA2 that stabilizes in adulthood. In this study, we focused on AMPAR composition and synaptic delivery in adult *Fmr*1 KO mice. Despite no differences in total GluA2 levels in adult *Fmr1* KO and WT brains, the amount of synaptic membrane-bound GluA2-containing AMPARs differs. Similarly, the total level of GluA1 or GluA3 AMPAR subunits was unchanged in *Fmr1* KO synaptoneurosomes, but the delivery of GluA1 and GluA3 to the synapse in response to stimulation was impaired in *Fmr1* KO. It is important to remember that synaptic strength is an alteration of the number and composition of AMPARs in the postsynaptic density; therefore, the real number of functional AMPARs in the postsynaptic membrane.

As we studied this phenomenon in adult mouse brain tissue, we observed changes that were initially triggered by the absence of FMRP but were stabilized over the course of development into adulthood. Thus, the observed deficits reflect both the direct effects of FMRP loss and compensatory mechanisms. For example, NMDAR-dependent long-term potentiation (LTP) is the result of increased AMPAR density in the PSD, and incorporation of GluA1-containing AMPAR is required for LTP initiation (Malinow and Malenka, 2002; Sheng and Kim, 2002; Shepherd and Huganir, 2007). AMPAR-mediated Ca^2+^ influx serves as the trigger for the induction of LTP or enhanced synaptic efficacy. Thus, the observed deficiency in synaptic delivery of AMPARs may help explain the LTP impairments previously observed/reported in *Fmr1* KO mice (Li et al., 2002; Larson et al., 2005). On the other hand, Ca^2+^-permeable AMPAR (CP-AMPAR)-dependent synaptic plasticity is a self-regulating mechanism, namely, repetitive activation of CP-AMPARs causes a rapid reduction in Ca^2+^ permeability by limiting the number of synaptic CP-AMPARs and incorporation of GluA2-containing Ca^2+^-impermeable AMPARs (CI-AMPAR), thus scaling down synaptic activity (Liu and Cull-Candy, 2000; Liu and Zukin, 2007). In this regard, the enhancement of GluA2-containing CI-AMPARs may reflect an attempt to decrease synaptic activity in *Fmr1* KO synapses. A wide range of studies showed neuronal and circuit hyperexcitability and sensory hypersensitivity in FXS individuals and the *Fmr1* KO model (Liu et al., 2021). This hyperactivity may result from aberrant activity-dependent plasticity mechanisms in early postnatal development when the neuronal networks are established. We hypothesize that described synaptic alterations such as the upregulation of GluA2-containing CI-AMPARs and lack of activity-induced incorporation of GluA1-3-containing AMPARs may in fact be a compensatory mechanism acting to stabilize circuit activity.

## Data availability statement

The original contributions presented in the study are publicly available. This data can be found here: ProteomeXchange Consortium via the PRIDE partner repository with the dataset identifier PXD043700.

## Ethics statement

The animal study was approved by 1st Local Ethical Committee for Experiments on Animals based at the Faculty of Biology, University of Warsaw, Miecznikowa Street 1, 02-096 Warsaw. The study was conducted in accordance with the local legislation and institutional requirements.

## Author contributions

MC: Formal analysis, Investigation, Validation, Visualization, Writing—review & editing. AB: Formal analysis, Investigation, Validation, Visualization, Writing—review & editing. MM: Formal analysis, Investigation, Validation, Visualization, Writing—review & editing. AS: Investigation, Validation, Writing—review & editing, Formal analysis. DC: Formal analysis, Validation, Writing—review & editing. JM: Investigation, Validation, Writing—review & editing. MD: Conceptualization, Funding acquisition, Supervision, Validation, Writing—review & editing. BK: Conceptualization, Formal analysis, Investigation, Supervision, Validation, Visualization, Writing—original draft.

## Supplementary material

Supplementary Figure S1 Supplementary Figure S1, relative to Figure 2. Total GluA1-3 levels in WT and *Fmr1* KO synaptoneurosomes after NMDA-R stimulation. Analysis of total GluA1, GluA2, and GluA3 protein levels (aliquots of the same samples as in Figure 2B, but without BS3-crosslinking) in WT and *Fmr1* KO SNs did not reveal any significant changes in AMPAR subunits among the two genotypes or in response to the stimulation (RM two-way ANOVA, *post-hoc* Sidak's or Tukey's multiple comparisons tests respectively; *p* > 0.05).
