# Supplementary figures and images for: Impaired synaptic incorporation of AMPA receptors in a mouse model of fragile X syndrome
Source: Front Mol Neurosci. 2023 Nov 9;16:1258615. doi: 10.3389/fnmol.2023.1258615 (PMC10665894; doi:10.3389/fnmol.2023.1258615)

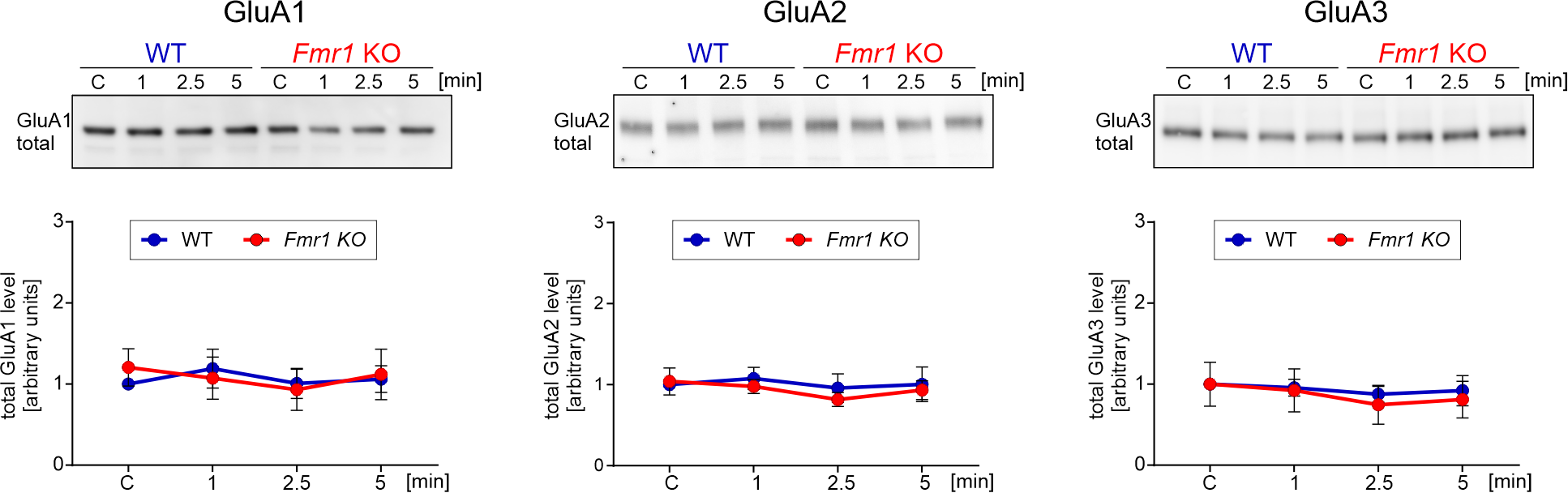

Supplement: Supplementary Figure S1 — , relative to Figure 2. Total GluA1-3 levels in WT and Fmr1 KO synaptoneurosomes after NMDA-R stimulation. Analysis of total GluA1, GluA2, and GluA3 protein levels (aliquots of the same samples as in Figure 2B, but without BS3-crosslinking) in WT and Fmr1 KO SNs did not reveal any significant changes in AMPAR subunits among the two genotypes or in response to the stimulation (RM two-way ANOVA, post-hoc Sidak's or Tukey's multiple comparisons tests respectively; p > 0.05). [file Image_1.tif]
